# Circulating Levels of the Interferon-γ-Regulated Chemokines CXCL10/CXCL11, IL-6 and HGF Predict Outcome in Metastatic Renal Cell Carcinoma Patients Treated with Antiangiogenic Therapy

**DOI:** 10.3390/cancers13112849

**Published:** 2021-06-07

**Authors:** Emilio Esteban, Francisco Exposito, Guillermo Crespo, Julio Lambea, Alvaro Pinto, Javier Puente, Jose A. Arranz, Miriam Redrado, Cristina Rodriguez-Antona, Carlos de Andrea, Marta Lopez-Brea, Esther Redin, Angel Rodriguez, Diego Serrano, Jorge Garcia, Enrique Grande, Daniel Castellano, Alfonso Calvo

**Affiliations:** 1 Medical Oncology Department, Hospital Central de Asturias, 33011 Oviedo, Spain; eestebang@seom.org; 2 IDISNA and Program in Solid Tumors, Center for Applied Medical Research (CIMA), University of Navarra, 31008 Pamplona, Spain; fexposito@unav.es (F.E.); miredrado@unav.es (M.R.); eredin@alumni.unav.es (E.R.); dserrano@unav.es (D.S.); 3 Department of Pathology, Anatomy and Physiology, School of Medicine, University of Navarra, 31008 Pamplona, Spain; ceandrea@unav.es; 4 CIBERONC, Instituto de Salud Carlos III (ISCIII), 28020 Madrid, Spain; 5 Medical Oncology Department, Burgos University Hospital, 09006 Burgos, Spain; gcrespo@saludcastillayleon.es; 6 Medical Oncology Department, University Hospital Lozano Blesa, 50009 Zaragoza, Spain; jjlambea@salud.aragon.es; 7 Medical Oncology Department, University Hospital La Paz, IdiPAZ, 28046 Madrid, Spain; alvaro.pinto@salud.madrid.org; 8 Medical Oncology Department, San Carlos Hospital, IdISSC, 28040 Madrid, Spain; javier.puente@salud.madrid.org; 9 Medical Oncology Department, University Hospital Gregorio Marañón, 28009 Madrid, Spain; joseangel.arranz@salud.madrid.org; 10 Hereditary Endocrine Cancer Group, Spanish National Cancer Research Center (CNIO), 28029 Madrid, Spain; crodriguez@cnio.es; 11 Department of Pathology, University Clinic of Navarra, 31009 Pamplona, Spain; 12 Medical Oncology Department, Marqués de Valdecilla Hospital, 39008 Santander, Spain; martafrancisca.lopezbrea@scsalud.es; 13 Medical Oncology Department, León Hospital, 24008 León, Spain; arodriguezsanchez@saludcastillayleon.es; 14 Medical Oncology Department, CHUS, 15706 Santiago de Compostela, Spain; jorgejgglez@yahoo.es; 15 Medical Oncology Department, MD Anderson Cancer Center Madrid, 28033 Madrid, Spain; egrande@mdanderson.es; 16 Medical Oncology Department, 12 de Octubre Hospital, 28041 Madrid, Spain; daniel.castellano@salud.madrid.org

**Keywords:** renal cell carcinoma, biomarkers, chemokines, cytokines, antiangiogenic therapy

## Abstract

**Simple Summary:**

We have studied blood levels of cytokines/chemokines in patients with metastatic renal cell carcinoma treated with sunitinib or pazopanib, with the goal of identifying biomarkers that can predict efficacy and survival. We have found that high levels of CXCL10, CXCL11, HGF and IL-6 before treatment associate with poor prognosis in these patients. Moreover, these factors are correlated in patients with renal carcinoma, suggesting a coordinated expression and secretion. We have developed a prognostic signature including these factors that predicts very accurately prognosis. Our results may help defining better the group of renal cell carcinoma patients who may benefit from sunitinib/pazopanib.

**Abstract:**

Sunitinib and pazopanib are standard first-line treatments for patients with metastatic renal cell carcinoma (mRCC). Nonetheless, as the number of treatment options increases, there is a need to identify biomarkers that can predict drug efficacy and toxicity. In this prospective study we evaluated a set of biomarkers that had been previously identified within a secretory signature in mRCC patients. This set includes tumor expression of c-Met and serum levels of HGF, IL-6, IL-8, CXCL9, CXCL10 and CXCL11. Our cohort included 60 patients with mRCC from 10 different Spanish hospitals who received sunitinib (*n* = 51), pazopanib (*n* = 4) or both (*n* = 5). Levels of biomarkers were studied in relation to response rate, progression-free survival (PFS) and overall survival (OS). High tumor expression of c-Met and high basal serum levels of HGF, IL-6, CXCL11 and CXCL10 were significantly associated with reduced PFS and/or OS. In multivariable Cox regression analysis, CXCL11 was identified as an independent biomarker predictive of shorter PFS and OS, and HGF was an independent predictor of reduced PFS. Correlation analyses using our cohort of patients and patients from TCGA showed that HGF levels were significantly correlated with those of IL-6, CXCL11 and CXCL10. Bioinformatic protein–protein network analysis revealed a significant interaction between these proteins, all this suggesting a coordinated expression and secretion. We also developed a prognostic index that considers this group of biomarkers, where high values in mRCC patients can predict higher risk of relapse (HR 5.28 [2.32–12.0], *p* < 0.0001). In conclusion, high plasma HGF, CXCL11, CXCL10 and IL-6 levels are associated with worse outcome in mRCC patients treated with sunitinib or pazopanib. Our findings also suggest that these factors may constitute a secretory cluster that acts coordinately to promote tumor growth and resistance to antiangiogenic therapy.

## 1. Introduction

Renal cell carcinoma (RCC) represents the seventh most common malignancy in men and the ninth in women [1]. From the different histological types of RCC, clear cell carcinoma is by far the most abundant (75–80% of the cases). According to 2018 GLOBOCAN data, an estimated 403,000 people a year are diagnosed with RCC, causing approximately 175,000 deaths yearly [2]. Up to 2005, the standard treatment for metastatic (m)RCC was the administration of interleukin 2 (IL-2) or interferon alpha (IFN-α), but less than 15% of the patients had a durable response [3]. Sorafenib, sunitinib, pazopanib and IFN-α+bevacizumab were later on introduced for the treatment of mRCC patients [4]. Both sunitinib and pazopanib exert their antiangiogenic effects by inhibiting signaling mediated by VEGFRs, PDGFRs, and c-Kit [4]. Available treatment options have now dramatically increased, with the approval of new tyrosine kinase inhibitors (TKIs) axitinib, cabozantinib, lenvatinib and tivozanib; mTOR inhibitors everolimus and temsirolimus; and immune checkpoint inhibitors (ICIs) nivolumab and ipilimumab [3,5]. Moreover, the therapeutic efficacy demonstrated for the combination therapies nivolumab+ipilimumab [6], avelumab+axitinib [7] and pembrolizumab+axitinib [8] has expanded the treatment landscape for mRCC even further.

Among this rapidly evolving armamentarium, sunitinib and pazopanib remain as possible recommended options for first-line treatment of patients with favorable-risk RCC according to the International Metastatic RCC Database Consortium (IMDC) [5]. Choosing the optimal drug and treatment sequence has become increasingly complex in the clinical practice and predictive biomarkers could be of great help for decision making [5]. Several predictive biomarkers, mainly related to angiogenesis pathways, have been identified for sunitinib and pazopanib. Blood baseline levels of VEGF, soluble (s)VEGFR2, (s)VEGFR3, IL-6 and single-nucleotide polymorphysms (SNPs) of *VEGF/VEGFR* and *IL-8* genes, among others, have been associated with response and outcome in RCC patients treated with sunitinib and/or pazopanib [9].

Selection of prognostic or predictive biomarkers usually relies on genetic alterations and secretory factors released by the cancer cells and the cells of the tumor microenvironment (TME). The *MET* gene codes for a membrane-anchored tyrosine kinase receptor that binds to its ligand hepatocyte growth factor (HGF), which is produced predominantly by stromal cells [10]. Activation of the HGF/c-Met axis initiates MAPK and AKT signalling pathways that promote proliferation and survival. Overexpression of c-Met in mRCC patients has been associated with an aggressive phenotype, increased risk of lymph node metastasis and an unfavorable outcome [11]. Peltola et al. described that high c-Met tumor levels in mRCC patients treated with sunitinib were associated with worse PFS and OS [12]. However, Kammerer-Jacquet et al. found no association with outcome [13].

**Figure 1:**
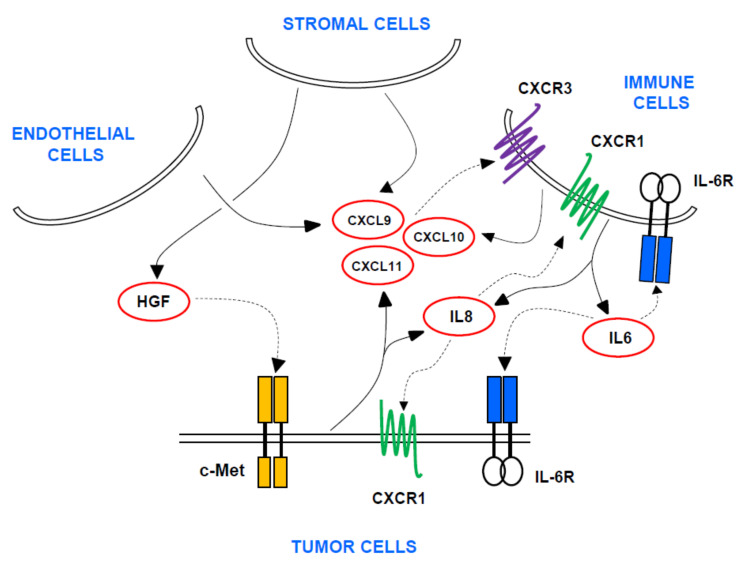
Scheme of biomarkers selected to study association with response, prognosis and toxicity in mRCC patients treated with sunitinib and/or pazopanib. The interactions between c-Met and HGF expression and activity of cytokines (IL-6, IL-8) and chemokines (CXCL9, CXCL10 and CXCL11) suggested by the literature, are shown with arrows. Tumor, endothelial, stromal and immune cells participate in this signaling network.

Overexpression of c-Met modifies the RCC TME, with increase in VEGF-mediated angiogenesis and PD-L1 expression [11,13]. In addition to enhancing tumor vascularity and permeability, VEGF exerts immunosuppression by intratumoral accumulation of myeloid-derived suppressor cells (MDSC) and regulatory T cells (Tregs), as well as by impeding the migration of cytotoxic T lymphocytes towards the TME [14,15]. Chemokines produced by the tumor cells and the TME are main mediators of immune cell recruitment, with a pro-inflammatory or anti-inflammatory effect depending on the chemokine and cancer type [16]. Polimeno et al. performed a high-throughput multiplex analysis of cytokines/chemokines together with a multiparametric flow cytometry analysis of immune cells in peripheral blood, using a cohort of 77 RCC patients and 40 healthy controls [17]. They found that HGF, VEGF, CXCL10, CXCL11, IL-8, IL-4, IL-6, IL-10 and G-CSF levels were higher in RCC patients in comparison with controls. Moreover, a protein network analysis revealed that these proteins were highly interconnected. In addition, the number of circulating CD4+/CD25^high^/FOXP3+ Tregs was higher in RCC patients, all this suggesting an immunosuppressive phenotype. Several other studies have shown that blood levels of IL-8 and IL-6 are increased and are associated with poor prognosis in RCC patients, as well as their role in promoting angiogenesis and suppressing the cytotoxic effect of immune cells [18,19]. The interferon-γ-induced chemokines CXCL9, CXCL10 and CXCL11 are CXCR3 ligands that regulate immune cell trafficking, differentiation and activation [16]. Although they exhibit pleiotropic roles in orchestrating tumor immunity and angiogenesis depending on the context, studies in RCC patients have shown that high tumor levels predict poor prognosis [20]. Based on this information we hypothesized that a network of probably interconnected cytokines/chemokines including CXCL9, CXCL10, CXCL11, IL-8 and IL-6 would have a relationship with the HGF/c-Met axis and would associate with survival in mRCC patients treated with the antiangiogenic drugs sunitinib or pazopanib. Our hypothesis is graphically depicted in Figure 1.

## 2. Patients and Methods 

### 2.1. Study Design and Patients

This was a prospective and multicentric study that included patients from 10 Spanish Institutions to identify biomarkers of response to sunitinib and pazopanib in mRCC patients as a first-line treatment, related to HGF/c-Met signalling and angiogenic/inflammatory TME. The primary endpoint was the association between biomarkers and progression-free survival (PFS) and overall response rate (ORR), according to Response Evaluation Criteria In Solid Tumors (RECIST) 1.1 criteria. The secondary endpoint was the association with overall survival (OS). PFS was defined as the time between treatment initiation and objective progression or death, and OS as the time elapsed between treatment initiation and the date of cancer-related death.

Inclusion criteria were as follows: patients ≥18 years of age, with locally advanced metastatic renal cell carcinoma, histopathologic diagnosis of renal clear-cell carcinoma, measurable disease by CT or MRI, life expectancy higher than 3 months and written informed consent. Patients with any other prior treatment, including cytokines, were excluded. The study was approved by the Ethics Committee at each institution and all participants provided written informed consent.

Patients received either 50 mg sunitinib daily for 4 weeks, followed by 2 weeks off the drug, or 800 mg pazopanib daily. In the case of 5 subjects, treatment of one of the drugs was discontinued at some point during the study (due to toxicity or other non-specified reasons) but patients received the other drug until progression. This group is referred to as sunitinib/pazopanib. All patients received treatment until there was evidence of disease progression, unacceptable toxicity, noncompliance with treatment, withdrawal of the patient’s informed consent or it was decided by the investigator.

### 2.2. Immunohistochemistry in Tumor Samples

A total number of 31 available paraffin-embedded tumor samples from primary or metastatic sites were obtained from patients included in our study. A tissue microarray (TMA) was built with 2 cores (1.5 mm diameter) per each paraffin-embedded sample. Immunohistochemistry was performed with the anti-c-Met antibody clone ab39075 at 1:100 (Abcam). Target Retrieval Solution (K8004, Dako) was used for antigen retrieval. The TMA was scanned using a Vectra Polaris slide scanner (Akoya Biosciences, Menlo Park, CA, USA) and the H-score was calculated.

### 2.3. Analysis of Circulating Cytokines/Chemokines in Blood

An exploratory study of biomarkers related to the Hepatocyte growth factor/c-Met (HGF/c-Met) axis, as well as the angiogenic/inflammatory factors interleukin-6 (IL-6), interleukin-8 (IL-8), monokine induced by interferon-gamma (IFN-γ) (MIG, or CXCL9), IFN-γ-induced protein 10 (IP-10 or CXCL10) and IFN-γ-inducible T-cell alpha chemoattractant (I-TAC or CXCL11) was conducted.

Serum samples were obtained from 10 mL blood collected at baseline and 3 months after treatment initiation. Samples were aliquoted and frozen at −80 °C until use. The Luminex’s xMAP^®^ Technology multiplex system was used to quantify simultaneously all these markers. Analysis was carried out with the Milliplex ™ MAP kit (Merck, Millipore Corporation, Kenilworth, NJ, USA), following the manufacturer-specific protocol. Concentration of each analyte was assessed according to standard calibration curves. Data are given in pg/mL.

### 2.4. Single Nucleotide Polymorphisms (SNPs) in Whole Blood Samples

Blood was collected in tubes with EDTA and aliquoted at −20 °C until use. Total DNA was obtained with the Maxwell^®^ RSC Blood DNA Kit (Promega) following the manufacturer’s protocol. Based on previous reports [21,22], the rs1176221 *MET* polymorphism was assessed. Genotyping was carried out using KASPar Technology (LGC Group; Hoddesdon, UK) in an ABI PRISM 7900HT Sequence Detection System (Applied Biosystems; Carlsbad, CA, USA).

### 2.5. Bioinformatic and Statistical Analysis

RNASeq2 level III RSEM data from RCC patients included in the international initiative “The Cancer Genome Atlas” (TCGA), were obtained using the Bioconductor package TCGA2STAT. Analyses were performed with the Log_2_ transformed RSEM values.

For the study of biomarkers in our cohort of patients, sample size calculation was estimated based on a pivotal phase III trial and a response rate of 40% [23]. Assuming 10% dropout rate, 80% power and 5% risk alpha, the sample size would correspond to 53 patients. Overall response rate (ORR) (complete response [CR] + partial response [PR]) was reported using descriptive statistics with 95% confidence intervals (CI). Progression-free survival (PFS) and overall survival (OS) were estimated using the Kaplan–Meier method. High or low levels of the biomarkers were established based on the median. To evaluate the effect of each biomarker on endpoints, Cox’s proportional hazards regression was used. Comparisons of circulating levels of cytokines/chemokines between responders and non-responders at baseline and 3 months post-treatment were performed with the Mann–Whitney U test. Correlation between biomarkers was evaluated by the Spearman’s test. We also generated a prognostic index (PI) including levels of HGF, IL-6, CXCL10 and CXCL11, using Cox’s regression analysis. The formulated PI was defined as the sum of the products of the B coefficient for each cytokine and its expression value (pg/mL). The discriminative ability of the PI was assessed by the use of Harrell’s concordance coefficient (C-index).

SPSS15.0 (Madrid, Spain), STATA/IC 12.1 (College Station, TX, USA) and GraphPad Prism 5 software (GraphPad) were used for statistical analysis and/or representation of the results. Data are expressed as means ± SD, statistical assessments were two-sided and *p*-values < 0.05 were deemed significant.

## 3. Results

### Patient Characteristics and Treatment Efficiency

**Figure 2:**
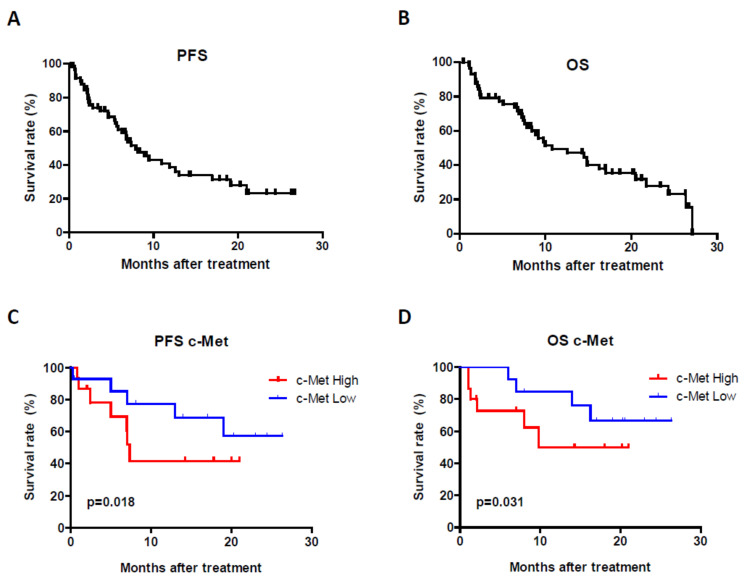
PFS (**A**) and OS (**B**) in our cohort of mRCC patients. c-Met protein expression in primary tumors or metastasis samples and its relationship with outcome (**C**). High levels of c-Met (above the H-score median) are associated with lower PFS (**C**) (*p* = 0.018) and OS (**D**) (*p* = 0.031). The Log-rank test was used for the analysis.

A total of 60 patients were enrolled in the study, with a median age of 65.17 years. Clinicopathological characteristics of the patients are shown in Table 1. Forty-six subjects were males and 14 were females. Sunitinib was administered as a first-line treatment in 51 patients (85%), pazopanib in 4 patients (6.7%) and both drugs during the course of their disease in 5 patients (8.3%, sunitinib/pazopanib group). In the population evaluable for efficiency endpoints (*n* = 51), the median PFS was 7.9 months (95% CI [5.0–10.7]) and the OS 14.8 months (95% CI [9.8–19.8]) (Figure 2A,B). Regarding response rates, 0 patients attained a full response, 20 (39.2%) had partial response, 20 (39.2%) showed stable disease and 11 (21.6%) progressed. The rate of clinical benefit was 78.4% (*n* = 40).

Table 2 shows a summary of toxicities found in our study. Full details of toxicity are shown in Supplementary Table S1. Almost all patients (96.7%) had some degree of toxicity throughout the study. The most common ones were asthenia (*n* = 38, 63.3%), mucositis (*n* = 33, 55%), diarrhea (*n* = 23, 38.3%), hypertension (*n* = 23, 38.3%) and thrombocytopenia (*n* = 18, 30%). Mucositis was found in all patients treated with sunitinib/pazopanib and in more than 50% of the patients who received sunitinib alone. No grade 4 toxicities were observed except for one patient treated with sunitinib who developed severe anemia.

## 4. Biomarkers in Relation to Efficiency Endpoints

### 4.1. c-Met Protein Expression in Tumors

We first evaluated c-Met protein expression in primary tumors or metastasis samples from available patients (*n* = 31). After immunohistochemical staining and calculation of the H-score, the median was established as a cut-off value to consider high or low c-Met protein expression for survival studies (Figure 2C,D). The logrank test showed that high c-Met protein levels were associated with lower PFS (*p* = 0.018) and OS (*p* = 0.031).

### 4.2. Circulating Cytokines, Chemokines and Growth Factors

**Figure 3:**
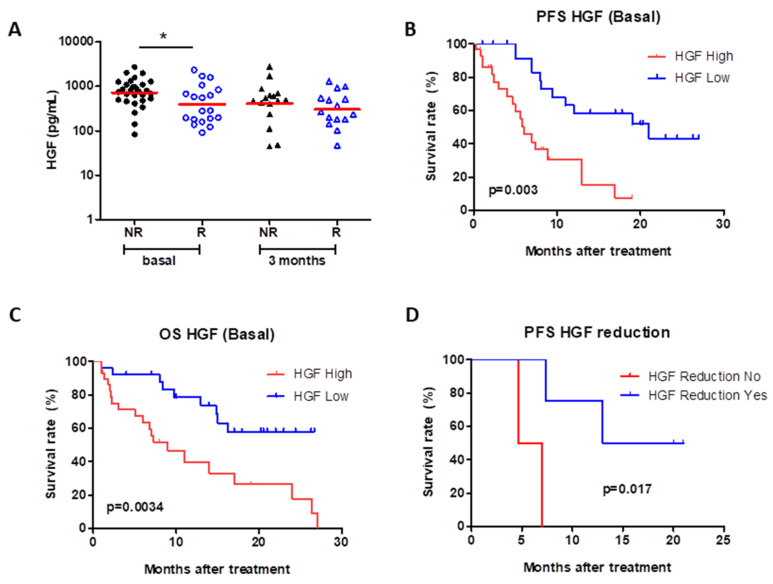
Blood levels of HGF and relationship with response and outcome. At baseline, levels of HGF are higher (*p* < 0.05) in patients without response (NR, *n* = 30) when compared to responders (R, *n* = 19). No changes were observed 3 months after treatment (NR, *n* = 16; R, *n* = 15) (**A**). HGF levels above 649.1 pg/mL are significantly associated with lower PFS (**B**) (*p* = 0.003) and OS (**C**) (*p* = 0.0034). A reduction in HGF levels after treatment is also significantly associated with lower PFS (**D**) (*p* = 0.017). The logrank test was used for the analysis.

At baseline, serum levels of the c-Met ligand HGF were higher (*p* < 0.05) in patients who did not respond (NR) to the treatment in comparison with those who did respond (R) (Figure 3A). Such a difference was not observed 3 months after treatment. Moreover, high basal HGF levels (over 649.1 pg/mL) were significantly associated with both reduced PFS (*p* = 0.003) and OS (*p* = 0.0034) (Figure 3B,C). A reduction in HGF levels over the course of treatment was also shown to be significantly associated with lower PFS (*p* = 0.017) (Figure 3D), but not with OS.

**Figure 4:**
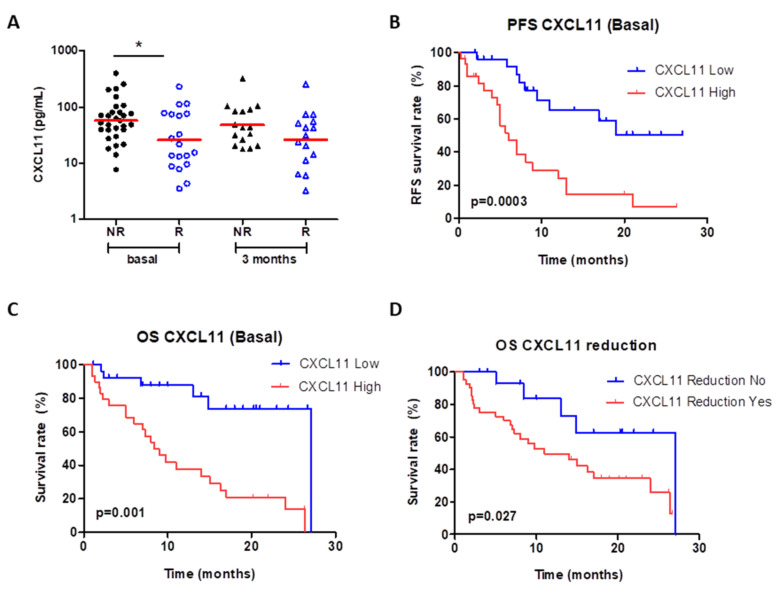
Blood levels of CXCL11 (ITAC). Higher basal levels are shown in non-responders (NR, *n* = 30, *p* < 0.05) than in responders (R, *n* = 19) with a similar trend 3 months after treatment (NR, *n* = 16; R, *n* = 15) (**A**). Elevated levels of this chemokine are significantly associated with decreased PFS (*p* = 0.003, (**B**)) and OS (*p* = 0.001, (**C**)). Reduction in CXCL11 over the course of drug administration is also related to worse OS (*p* = 0.027, (**D**)). The logrank test was used for the analysis.

Levels of CXCL11 (I-TAC) were significantly higher in patients who did not respond to treatment (*p* < 0.05) than in those who responded (Figure 4A). Such a trend was also observed 3 months after treatment, but differences were not statistically significant (Figure 4A). High levels of CXCL11 (above 39.4 pg/mL) were significantly associated with reduced PFS (*p* = 0.0003) and OS (*p* = 0.001) (Figure 4B,C). In addition, in patients in which a reduction in CXCL11 levels 3 months after treatment was observed, significantly lower OS (*p* = 0.027) (Figure 4D), but not PFS, was found.

**Figure 5:**
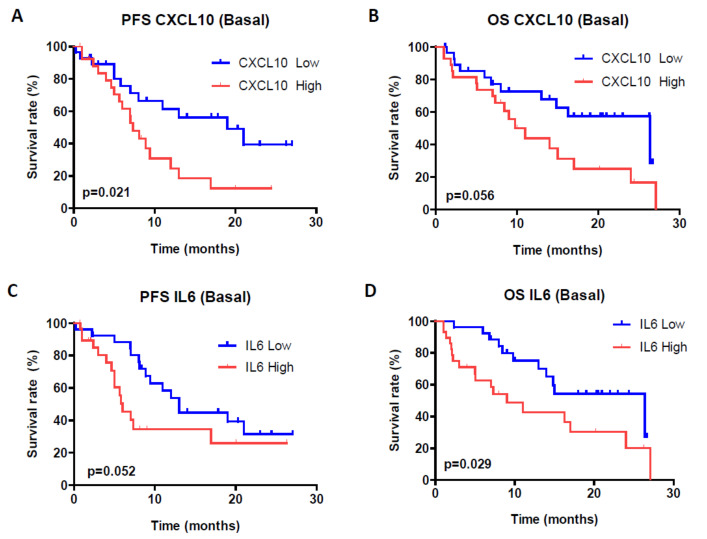
Blood levels of CXCL10 (IP-10) and IL-6. Both PFS (**A**) and OS (**B**) are significantly shorter in patients with high basal levels of CXCL10. Patients with high IL-6 basal concentration tend to show lower PFS (*p* = 0.052) (**C**) and are statistically reduced in the case of OS (*p* = 0.029) (**D**).

High levels of CXCL10 (IP-10) in patients (higher than 208.4 pg/mL) before drug administration were also able to predict reduced PFS (*p* = 0.021) (Figure 5A), whereas borderline statistical significance was observed in the case of OS (*p* = 0.056) (Figure 5B). No differences in survival rates were demonstrated 3 months post-treatment. Levels of CXCL9 (MIG) were not associated with response or survival.

Regarding IL-6, slightly lower levels were found in patients who responded to the drugs, before treatment and 3 months post drug administration, but without statistical differences between the groups (Supplementary Figure S1A). In survival analyses we observed that high basal levels of IL-6 (over 6.0 pg/mL) were significantly associated with lower OS (*p* = 0.029) and were almost significantly associated with lower PFS (*p* = 0.052) (Figure 5C,D). On the contrary, no relationship between levels of IL-8 and any of the efficiency parameters was observed.

### 4.3. Cox Proportional-Hazards Model and Prognostic Index for Risk Stratification

Using the univariable Cox proportional-hazards model, high HGF, CXCL10 and CXCL11 levels were shown to be significantly related to lower PFS and OS, except for CXCL10 in the case of OS (Table 3 and Table 4). Multivariable Cox analysis considering variables HGF, IL6, CXCL10 and CXCL11, revealed that CXCL11 was an independent biomarker to predict PFS (HR 3.23 [1.42–7.37], *p* = 0.005) and OS (HR 4.24 [1.53–11.70], *p* = 0.005). HGF was also shown as an independent biomarker in the prediction of PFS (HR 3.31 [1.43–7.65], *p* = 0.005) but not OS (HR 2.18 [0.90–5.23], *p* = 0.081). We next conducted a Cox regression analysis to develop a prognostic index (PI) that took into account protein levels of HGF, IL-6, CXCL10 and CXCL11. The PI equation was defined by the following formula: PI = 0.00036 × HGF + 0.0069 × IL-6 + 0.0004 × CXCL10 + 0.0063 × CXCL11.

As shown in Table 3 and Table 4, the PI was superior to individual biomarkers alone in predicting risk of PFS (HR 5.28 [2.32–12.0], *p* < 0.0001). Regarding OS, high levels of CXCL11 alone were able to predict lower survival better than any of the other biomarkers or the PI (HR 5.38 [2.04–14.19], *p* < 0.0001 for CXCL11 and HR 4.82 [2.03–11.42], *p* < 0.001 for the PI). For assessment of the possible influence of any of the clinicopathological characteristics of our cohort on the PI, we used Chi^2^ correlation after dichotomization (low vs. high) of the PI by the median. This analysis ruled out any significant influence of clinicopathological parameters and the variables studied (Supplementary Table S2). Performance, assessed by sensitivity, specificity and area under the receiver operating characteristic curve (ROC), were estimated for each individual marker and the PI (Supplementary Figure S2).

We also calculated Harrel’s concordance coefficient (C-index) for the PI: 0.70 for PFS and 0.69 for OS (Supplementary Table S3). The PI fulfilled the proportional hazards assumption for both RFS and OS (*p* = 0.52 and 0.73, respectively). Comparison of the C-index between the PI and individual biomarkers showed that the PI outperformed IL-6 and CXCL10 for predicting PFS and CXCL10 in the case of OS, with no statistical differences for the other markers (Supplementary Table S2).

### 4.4. Circulating HGF Levels Strongly Correlate with CXCL11, CXCL10 and IL-6 Levels

**Figure 6:**
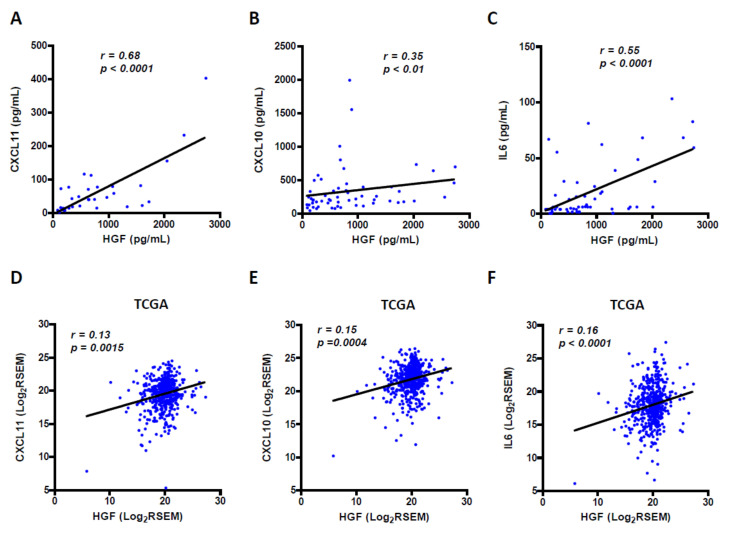
Correlation analysis between HGF levels and amount of CXCL11, CXCL10 and IL6. In our cohort of patients, a very significant positive correlation is observed between blood HGF and CXCL11 (*p* < 0.0001) (**A**), CXCL10 (*p* < 0.01) (**B**) and IL-6 (*p* < 0.0001) (**C**). Using public data from TCGA, mRNA levels of HGF are also significantly correlated with those of CXCL11 (*p* = 0.0015) (**D**), CXCL10 (*p* = 0.0004) (**E**) and IL-6 (*p* = 0.0001) (**F**). Correlation was determined using Spearman’s rank correlation test.

We next studied correlation between the HGF/c-Met axis and levels of CXCL11, CXCL10 and IL6. While no correlation was shown between c-Met tumor protein levels and circulating chemokines/cytokines, a very significant positive correlation was observed between its ligand HGF and CXCL11 (*p* < 0.0001), CXCL10 (*p* < 0.01) and IL-6 (*p* < 0.0001) (Figure 6A–C). We also studied whether tumor mRNA levels of these factors would be correlated as well. Using the TCGA dataset of RCC samples, we found that expression of HGF was also significantly correlated with expression of CXCL11 (*p* = 0.0015), CXCL10 (*p* = 0.0004) and IL-6 (*p* < 0.0001) (Figure 6D–F). All this suggests an interrelated expression and secretion of these factors in RCC patients.

Using the publicly available software STRING, protein–protein interactions between these cytokines/chemokines were evaluated (Supplementary Figure S1B). There was a significant interaction between these proteins (PPI enrichment *p* value: 0.0067), with a local cluster coefficient of 0.833.

### 4.5. Genetic Polymorphism rs1176221 in the MET Gene

We studied germline polymorphisms rs1176221 in *MET* gene in 57 of the patients where a whole blood sample was available. In 86% (*n* = 49) of the patients we found rs1176221=G/G, whereas in the remaining 14.0% (*n* = 8) we observed a G/A. The presence of the variant was not associated with any of the parameters related to efficiency studied: response to treatment, PFS or OS.

## 5. Biomarkers in Relation to Toxicity

No significant association between amount of cytokines/chemokines or a particular SNP and toxicity was found.

## 6. Discussion

At present, there is no conclusive evidence supporting one specific sequence for the treatment of mRCC patients, with numerous therapeutic options in first-, second- and third-line settings [9]. Even in the era of immunotherapy, anti-angiogenic therapy with sunitinib and pazopanib remains as an appropriate option as first-line treatment for patients with IMDC favorable risk [5]. In this scenario, biomarkers could help in deciding the optimal drug for a particular patient in terms of efficacy and toxicity. In the case of sunitinib and pazopanib, several biomarkers have been related to response, survival or side effects. Most of these biomarkers are related to VEGF and interleukin signaling [9]. In the present study we have shown that high basal circulating levels of HGF, CXCL10, CXCL11 and IL-6 are associated with a lack of response to sunitinib and/or pazopanib, and a worse outcome in mRCC patients. In keeping with these results, in a phase III clinical trial including RCC patients that received either pazopanib or placebo, high baseline serum concentration of IL-8, HGF, IL-6 and TIMP1 was significantly associated with worse prognosis, independently on the treatment arm [24].

In agreement with our initial hypothesis, HGF levels are strongly correlated with those of CXCL10, CXCL11 and IL-6, suggesting that HGF/c-Met signaling is inducing an angiogenic and inflammatory response that impairs the effect of these TKIs. Moreover, our bioinformatic analysis using the protein–protein interaction software STRING revealed a significant network that comprises these biomarkers. Such results are also in keeping with those reported by Polimeno et al. who identified a functional cluster of blood factors from mRCC patients that includes HGF, IL-6, CXCL10 and CXCL11, suggesting a functional coordinate secretion [17]. In addition, the prognostic index that we have developed shows that PFS in mRCC patients treated with TKIs can be more accurately predicted when all these biomarkers are considered rather than just taking into account single biomarkers.

c-Met signaling has been shown to remodel tumor vasculature and induce the production of IL-6 [25,26,27] and IL-8 [28,29]. Chronic exposure of these pro-inflammatory cytokines stimulates cancer cell proliferation, angiogenesis and recruitment of immune cells with an immunosuppressive profile [30]. During inflammation, tumor infiltrating immune cells produce INF-γ, which in turn activates a variety of INF-γ-induced genes through the JAK/STAT pathway, such as CXCL9, CXCL10 and CXCL11 [31].

The C-X-C motif chemokine ligand 10 (CXCL10) plays a role in chemotaxis, regulation of cell growth and angiostasis [32]. Deregulation of CXCL10 has been related to chronic inflammation, autoimmunity, tumor development, metastatic dissemination and infectious diseases [32]. Under pro-inflammatory conditions, CXCL10 can be secreted by monocytes, neutrophils, eosinophils, endothelial cells and fibroblasts in response to INF-γ and act on target cells through the CXCR3 receptor, expressed on activated T lymphocytes, natural killer (NK) cells, inflammatory dendritic cells, macrophages and B cells [32]. Both pro- and anti-tumor effects have been described for CXL10, depending on the tumor type and context [32]. CXCL10 is up-regulated in many cancer types including RCC, where very high levels compared with healthy individuals have been reported [20]. Levels of CXCL10 are modulated by drug treatment in mRCC patients. Thus, Choueiri et al. [33] described that CXCL10 increased by 37% in blood samples from mRCC patients treated with nivolumab. Xu et al. [34] have recently conducted an analysis of circulating biomarkers in plasma samples from patients that participated in the ECOG-ACRIN 2805 (ASSURE) trial. In this study, consisting of adjuvant treatment with VEGFR TKIs in resected mRCC patients, CXCL10 was demonstrated to be significantly elevated 4–6 weeks post treatment in those subjects receiving sunitinib or sorafenib. Moreover, high basal levels of this chemokine were associated with reduced DFS [34]. Our results are also in line with these findings, strongly suggesting that CXCL10 warrants further consideration as a promising biomarker of response to sunitinib.

CXCL11, also known as interferon-γ-inducible T-cell chemoattractant alpha (I-TAC), is expressed by different cell types, including leukocytes, and is responsible for activation and accumulation of T cells upon binding to CXCR3. An immunohistochemical study in RCC samples found a 14.9-fold increase in CXCl11 levels compared to normal kidneys [35]. Double immunofluorescence identified this chemokine in pericytes and vascular smooth muscle cells (VSMCs) of tumor angiogenic vessels [35]. Similar to CXCL10, CXCL11 may play anti-tumor or pro-tumor functions depending on the tissue context [16]. In experimental models of colon cancer, local exposure to CXCL11 stimulates tumor growth, without affecting tumor angiogenesis in vivo [36], but no information is available for RCC models. The role of CXCL11 as a biomarker in RCC patients has also been poorly studied and our findings showing an association with a response to sunitinib and/or pazopanib may encourage future studies to evaluate this chemokine as a possible predictor of response to TKIs.

In the evolving landscape of treatment options for patients with RCC, the TKI cabozantinib showed superiority over sunitinib in IMDC intermediate- and poor-risk patients (CABOSUN trial [37]). Cabozantinib targets VEGF receptors in addition to c-Met and AXL, which are involved in resistance against anti-VEGF therapies [38]. Although predictive biomarkers for cabozantinib have not been identified yet, the analysis of circulating HGF, IL-6, CXCL10 and CXCL11 could be appropriate candidates. This hypothesis is supported by results presented here and the publications suggesting that these factors may constitute a secretory cluster in RCC related to c-Met signaling.

Another interesting area that is currently being explored is the combination between ICIs and antiangiogenic TKIs. VEGF increases the number of Tregs and MDSCs in tumors, whereas antiangiogenic therapy using bevacizumab or sunitinib reduces these immunosuppressive populations [39]. The crosstalk between angiogenesis, inflammatory cytokines and chemotaxis of immune cells has fueled trials testing combinations between VEGF-TKI and anti-PDL-1/PD-1 in RCC patients [39]. These trials have shown promising anti-tumor efficacy [39], but biomarkers will be necessary to predict who may benefit from these treatments. Since CXCL10, CXCL11 and IL-6 are in the crossroad between angiogenesis and immunity, they may also be considered as potential biomarkers in this setting.

In summary, we have shown that high basal circulating levels of HGF, IL-6, CXCL10 and CXCL11 are associated with reduced PFS and OS in mRCC patients treated with sunitinib and/or pazopanib. IL-6, CXCL10 and CXCL11 levels are strongly correlated with HGF levels in these patients, advocating the existence of a secretory network of angiogenic and immunosuppressive factors related to c-Met signaling which may impair the effect of TKIs. Hence, these cytokines/chemokines could represent accurate biomarkers of response to antigangiogenic-based therapy. Nonetheless, our study has several limitations, such as the limited number of patients and the lack of confirmatory results in an independent cohort. Validation studies in a prospective series of patients should be performed to ascertain the possible clinical value of these biomarkers. In addition, it is suggestive to consider these biomarkers in RCC patients treated with immunomodulatory drugs.

## Figures and Tables

**Table 1:** Clinicopathological characteristics of the patients included in the study.

Corhort (*n* = 60)	
Gender	*n* (%)
Male	46 (76.7)
Female	14 (23.3)
Age at diagnosis (primary tumor)	
<65	31 (51.7)
≥65	29 (48.3)
Age at metastasis	
<65	30 (50)
≥65	30 (50)
Time between diagnosis and metastasis	
Metastasis at diagnosis	23 (38.3)
≤1 years	15 (25)
≤5 years	14 (23.3)
>5 years	8 (13.3)
Metastatic disease at study enrollment	60 (100)
Number of metastatic sites	
1	22 (36.7)
2	30 (50)
3	4 (6.7)
4	4 (6.7)
Risk factor (MSKCC *)	
Good	9 (15)
Intermediate	42 (70 )
Bad	9 (15)
Therapy	
Sunitinib	51 (85)
Pazopanib	4 (6.7)
Sunitib + Pazopanib	5 (8.3)

*, MSKCC = Memorial Sloan Kettering Cancer Centre.

**Table 2:** Summary of toxicity findings.

Toxicity	Sunitinib *n* (%)	Pazopanib *n* (%)	Sunitinib/Pazopanib *n* (%)	Total	*p*
Asthenia	33 (64.7)	3 (75.0)	2 (40)	38 (63.3)	0.495
Neutropenia	6 (11.8)	-	-	6 (10)	0.356
Hand-foot syndrome	14 (27.5)	-	1 (20)	15 (25.0)	0.282
Hypertension	17 (33.3)	2 (50.0)	1 (20.0)	20 (33.3)	0.635
Hypothyroidism	5 (9.8)	-	-	5 (8.3)	0.427
Cardiotoxicity	1 (2.0)	-	-	1 (1.7)	0.849
Mucositis	27 (52.9)	1 (25)	5 (100)	33 (55.0)	**0.023**
Diarrhea	18 (35.3)	3 (75.0)	2 (40.0)	23 (38.3)	0.297
Leukopenia	7 (13.7)	-	-	7 (11.7)	0.297
Anemia	14 (27.5)	-	-	14 (23.3)	0.073
Thrombocytope-nia	15 (29.4)	1 (25)	2 (40.0)	18 (30.0)	0.864

**Table 3:** Univariable and multivariable Cox proportional-hazards model for PFS.

Cytokine/Chemokine				Univariable Cox Hazards Model (PFS)			Multivariable Cox Hazards Model (PFS)				
	Cohort	Progression									
	N	N	Person-Years	Crude HR	95% CI	*p*	HR	95% CI	*p*	LR	*p*
HGF	55	28								22.3	0.0002
Low	27	10	0.24	1		0.001	1		0.005		
High	28	18	0.44	3.85	(1.74–8.47)		3.31	(1.43–7.65)			
IL-6	55										
Low	27	14	0.34	1		0.144	1		0.531		
High	28	14	0.34	1.73	(0.82–3.64)		1.28	(0.58–2.79)			
CXCL-10	55										
Low	27	10	0.24	1		0.026	1		0.852		
High	28	18	0.44	2.33	(1.10–4.94)		1.08	(0.46–2.52)			
CXCL-11	55										
Low	27	8	0.19	1		0.001	1		0.005		
High	28	20	0.48	3.86	(1.75–8.50)		3.23	(1.42–7.37)			
Prognostic Index (PI)	55										
Low	27	9	0.22	1		<0.0001					
High	28	19	0.46	5.28	(2.32–12.0)						

**Table 4:** Univariable and multivariable Cox proportional-hazards model for OS.

Cytokine/Chemokine				Univariable Cox Hazards Model (OS)			Multivariable Cox Hazards Model (OS)				
	Cohort	Exitus									
	N	N	Person-Years	Crude HR	95% CI	*p*	HR	95% CI	*p*	LR	*p*
HGF	55	28								19.29	0.0007
Low	27	8	0.16	1		0.005	1		0.081		
High	28	20	0.39	3.15	(1.40–7.07)		2.18	(0.90–5.23)			
IL-6	55										
Low	27	11	0.22	1		0.069	1		0.715		
High	28	17	0.34	1.99	(0.94–4.20)		1.16	(0.51–2.64)			
CXCL-10	55										
Low	27	10	0.20	1		0.062	1		0.942		
High	28	18	0.35	2.07	(0.96–4.46)		1.22	(0.39–2.37)			
CXCL-11	55										
Low	27	4	0.08	1		0.001	1		0.005		
High	28	22	0.43	5.38	(2.04–14.1)		4.24	(1.53–11.7 )			
Prognostic Index (PI)	55										
Low	27	7	0.14	1		<0.0001					
High	28	21	0.41	4.82	(2.03–11.42)						

## Data Availability

Data are available on request from the corresponding author.

## Supplementary Materials

The following are available online at https://www.mdpi.com/article/10.3390/cancers13112849/s1, Supplementary Figure S1. Blood levels of IL-6 in responder and non-responder patients (A). STRING network analysis reveals significant interactions between HGF, IL-6, CXCL10 and CXCL11 (PPI enrichment p value: 0.0067) (B). Supplementary Figure S2. Performance parameters for prediction of PFS and OS: ROC curves for the individual biomarkers and for the PI. Sensitivity and specificity data at the optimal cut-off value are shown in the Table below the graphics; Table S1. Toxicities found in the study, Table S2. Chi2 correlation results evaluating the Prognostic Index (PI) (low vs. high) and the clinicopathological variables; Table S3. Harrell’s Concordance (C) coefficient for each individual biomarker and for the PI index.
