# Supplementary material for: Circulating Levels of the Interferon-γ-Regulated Chemokines CXCL10/CXCL11, IL-6 and HGF Predict Outcome in Metastatic Renal Cell Carcinoma Patients Treated with Antiangiogenic Therapy
Source: Cancers (Basel). 2021 Jun 7;13(11):2849. doi: 10.3390/cancers13112849 (PMC8201218; doi:10.3390/cancers13112849)
Supplement: Supplementary file 1 [file cancers-13-02849-s001.zip › cancers-1174644-supplementary.pdf]

Supplementary Materials: Circulating Levels of the Interferon-γ-Regulated Chemokines CXCL10/CXCL11, IL-6 and HGF Predict Outcome in Metastatic Renal Cell Carcinoma Patients Treated with Antiangiogenic Therapy

Emilio Esteban, Francisco Exposito, Guillermo Crespo, Julio Lambea, Alvaro Pinto, Javier Puente, Jose Angel Arranz, Miriam Redrado, Cristina Rodriguez-Antona, Carlos de Andrea, Marta Lopez-Brea, Esther Redin, Angel Rodriguez, Diego Serrano, Jorge Garcia, Enrique Grande, Daniel Castellano and Alfonso Calvo

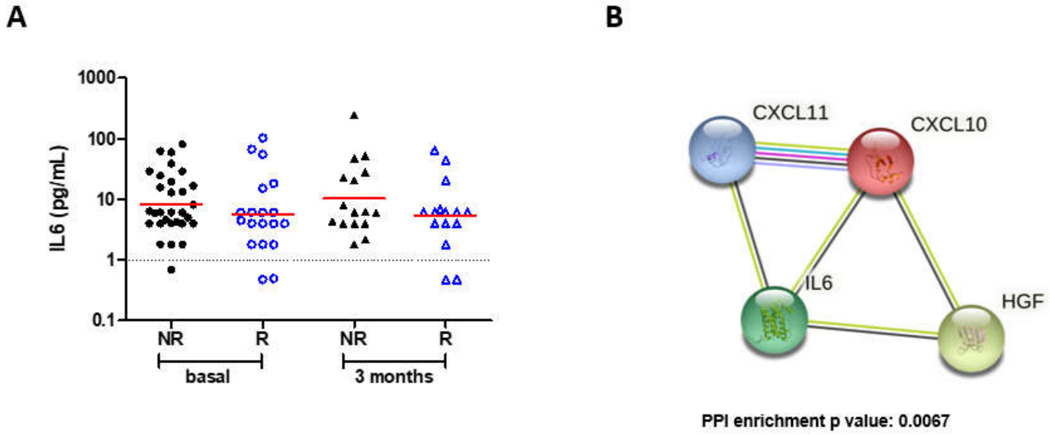

Figure S1. Blood levels of IL-6 in responder and non-responder patients (**A**). STRING network analysis reveals significant interactions between HGF, IL-6, CXCL10 and CXCL11 (PPI enrichment p value: 0.0067) (**B**).

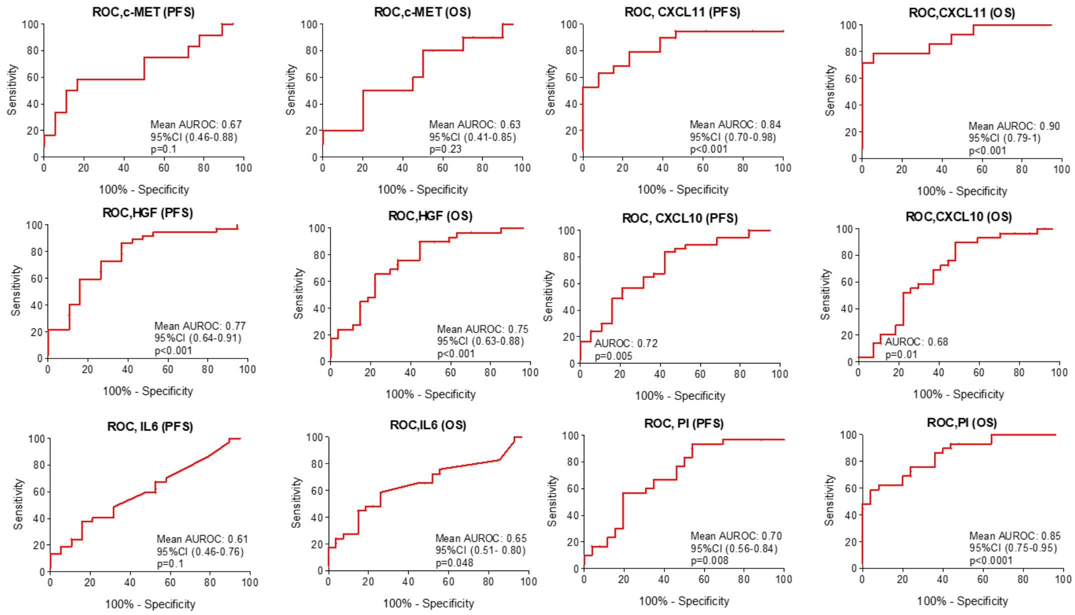

Supplementary Figure 2

Cytokine	cytokine cut-off value	Sensitivity%	95% CI	Specificity%	95% CI	Likelihood ratio
**HGF PFS**	> 661.8	64.86	47.46% to 79.79%	73.68	48.80% to 90.85%	2,46
**HGF OS**	> 661.8	68.97	49.17% to 84.72%	66.67	46.04% to 83.48%	2,07
**CXCL11 PFS**	> 39.64	73.68	48.80% to 90.85%	76.92	46.19% to 94.96%	3,19
**CXCL11 OS**	> 39.64	78.57	49.20% to 95.34%	66.67	40.99% to 86.66%	2,36
**CXCL10 PFS**	> 207.4	62.16	44.76% to 77.54%	68.42	43.45% to 87.42%	1,97
**CXCL10 OS**	> 207.4	65.52	45.67% to 82.06%	62.96	42.37% to 80.60%	1,77
**IL6 PFS**	> 6.040	59.46	42.10% to 75.25%	52.63	28.86% to 75.55%	1,26
**IL6 OS**	> 6.040	65,52	45.67% to 82.06%	55.56	35.33% to 74.52%	1,47
**PI PFS**	> 0.8532	66.67	49.82% to 86.25%	65.38	44.33% to 82.79%	2,03
**PI OS**	> 0.8532	75.86	56.46% to 89.70%	76.00	54.87% to 90.64%	3,16

Figure S2. Performance parameters for prediction of PFS and OS: ROC curves for the individual biomarkers and for the PI. Sensitivity and specificity data at the optimal cut-off value are shown in the Table below the graphics.

Table S1. Toxicities found in the study.

		**Toxicity**				***p***
Presented Toxicity	No	2 (39%)	-	-	2 (3.3)	0.718
	Yes	49 (96.1)	4 (100)	5 (100)	58 (96.7)	
**Toxicity (Grade)**		**Sunitinib n (%)**	**Pazopanib n (%)**	**Sunitinib / Pazopanib n (%)**	**Total**	***p***
Asthenia	G1	10 (30.3)	-	1 (50.0)	11 (28.9)	0.281
	G2	17 (51.5)	2 (66.7)	-	19 (50.0)	
	G3	6 (18.2)	1 (33.3)	1 (50.0)	8 (21.1)	
	G4	-	-	-	-	
	⚜	33 (64.7)	3 (75.0)	2 (40)	38 (63.3)	0.495
Neutropenia	G1	1 (16.7)	-	-	1 (16.7)	-
	G2	2 (33.3)	-	-	2 (33.3)	
	G3	3 (50.0)	-	-	3 (50.0)	
	G4	-	-	-	-	
	⚜	6 (11.8)	-	-	6 (10)	0.356
Hand-foot syndrome	G1	4 (28.6)	-	-	4 (26.7)	0.242
	G2	7 (50.0)	-	-	7 (46.7)	
	G3	3 (21.4)	-	1 (100)	4 (26.7)	
	G4	-	-	-	-	
	⚜	14 (27.5)	-	1 (20)	15 (25.0)	0.282
Hypertension	G1	9 (56.3)	1 (50.0)	-	10 (52.6)	0.121
	G2	6 (37.5)	-	-	6 (31.6)	

	G3	1 (6.3)	1 (50.0)	1 (100)	3 (15.8)	
	G4	-	-	-	-	
	⚜	17 (33.3)	2 (50.0)	1 (20.0)	20 (33.3)	0.635
Hypothyroidism	G1	2 (50.0)	-	-	2 (50.0)	-
	G2	2 50.0)	-	-	2 50.0)	
	G3	-	-	-	-	
	G4	-	-	-	-	
	⚜	5 (9.8)	-	-	5 (8.3)	0.427
Cardiotoxicity	G1	-	-	-	-	-
	G2	-	-	-	-	
	G3	1 (100)	-	-	1 (100)	
	G4	-	-	-	-	
	⚜	1 (2.0)	-	-	1 (1.7)	0.849
Mucositis	G1	13 (48.1)	1 (100)	2 (40.0)	16 (48.5)	0.805
	G2	10 (37.0)	-	2 (40.0)	12 (36.4)	
	G3	4 (14.8)	-	1 (20)	5 15.2)	
	G4	-	-	-	-	
	⚜	27 (52.9)	1 (25)	5 (100)	33 (55.0)	0.023
Diarrhea	G1	9 (52.9)	3 (100)	1 (50.0)	13 (59.1)	0.403
	G2	6 (35.3)	-	1 (50.0)	7 (31.8)	
	G3	2 (11.8)	-	-	2 (9.1)	
	G4	-	-	-	-	
	⚜	18 (35.3)	3 (75.0)	2 (40.0)	23 (38.3)	*0.297*
Leukopenia	G1	4 (57.1)	-	-	4 (57.1)	-
	G2	2 (28.6)	-	-	2 (28.6)	
	G3	1 (14.3)	-	-	1 (14.3)	
	G4	-	-	-	-	
	⚜	7 (13.7)	-	-	7 (11.7)	0.297
Anemia	G1	6 (42.9)	-	-	6 (42.9)	-
	G2	6 (42.9)	-	-	6 (42.9)	
	G3	1 (7.1)	-	-	1 (7.1)	
	G4	1 (7.1)	-	-	1 (7.1)	
	⚜	14 (27.5)	-	-	14 (23.3)	0.073
Thrombocytopeni a	G1	7 (50.0)	-	-	7 (41.2)	0.314
	G2	3 (21.4)	-	1 (50.0)	4 (23.5)	
	G3	4 (28.6)	1 (100)	1 (50.0)	6 (35.3)	
	G4	-	-	-	-	
	⚜	15 (29.4)	1 (25)	2 (40.0)	18 (30.0)	0.864

Table S2. Chi² correlation results evaluating the Prognostic Index (PI) (low vs. high) and the clinicopathological variables.

**Clinicopathological variables**	**PI Low n(%)**	**PI High n(%)**	***p***
Gender			
Male	21 (77.8)	20 (71.4)	0.589
Female	6 (22.2%)	8 (28.6)	
Age at diagnosis (primary tumor)			
<65	12 (42.9)	15 (55.6)	0.346
≥65	16 (57.1)	12 (44.4)	
Age at metastasis			
<65	12 (42.9)	15 (55.6)	0.346
≥65	16 (57.1)	12 (44.4)	
Time between diagnosis and metastasis			

Metastasis at diagnosis	11 (39.3)	9 (33.3)	0.655
≤1 years	6 (21.4)	9 (33.3)	
≤5 years	8 (28.6)	5 (18.5)	
>5 years	3 (10.7)	4 (14.8)	
Metastatic disease at study enrollment			
Number of metastatic sites			
1	9 (32.1)	11 (42.3)	0.086
2	17 (60.7)	9 (34.6)	
3	0 (0.0)	4 (15.4)	
4	2 (7.1)	2 (7.7)	
Risk factor (MSKCC*)			
Good	7 (25.9)	2 (7.4)	0.062
Intermediate	18 (66.7)	19 (67.9)	
Bad	2 (7.4)	7(25.0)	
Therapy			
Sunitinib	24 (85.7)	25 (89.3)	0.508
Pazopanib	1 (3.6%)	2 (7.1)	
Sunitib + Pazopanib	3 (10.7)	1 (3.6)	

*MSKCC = Memorial Sloan Kettering Cancer Centre.

Table S3. Harrell's Concordance (C) coefficient for each individual biomarker and for the PI index.

**Cytokine/ chemokine**	**PFS Harrell's C**	**95% CI**	***p***	**OS Harrell's C**	**95% CI**	***p***
HGF	0.68	(0.60-0.75)	<0.0001	0.65	(0.55–0.74)	<0.0001
IL–6	0.60	(0.49–0.69)	<0.0001	0.61	(0.51–0.72)	<0.0001
CXCL10	0.58	(0.48–0.69)	<0.0001	0.58	(0.47–0.68)	<0.0001
CXCL11	0.68	(0.60–0.76)	<0.0001	0.67	(0.57–0.76)	<0.0001
PI	0.70	(0.63–0.78)	<0.0001	0.69	(0.61–0.77)	<0.0001
**Comparison**	**Coefficient**	**95% CI**	***p***	**Coefficient**	**95% CI**	***p***
PI vs. HGF	0.026	(–0.06–0.11)	0.54	0.043	(–0.04–0.13)	0.337
PI vs. IL–6	0.108	(0.004–0.21)	0.041	0.074	(–0.02–0.17)	0.128
PI vs. CXCL10	0.117	(0.02–0.21)	0.015	0.110	(0.006–0.21)	0.037
PI vs CXCL11	0.021	(–0.04–0.08)	0.52	0.023	(–0.04–0.95)	0.52
